# State-dependent evolutionary models reveal modes of solid tumour growth

**DOI:** 10.1038/s41559-023-02000-4

**Published:** 2023-03-09

**Authors:** Maya A. Lewinsohn, Trevor Bedford, Nicola F. Müller, Alison F. Feder

**Affiliations:** 1 Department of Genome Sciences, University of Washington, Seattle, WA USA; 2 Vaccine and Infectious Disease Division, Fred Hutchinson Cancer Center, Seattle, WA USA; 3 Howard Hughes Medical Institute, Seattle, WA USA

**Keywords:** Phylogenetics, Cancer genetics, Cancer genetics

## Abstract

Spatial properties of tumour growth have profound implications for cancer progression, therapeutic resistance and metastasis. Yet, how spatial position governs tumour cell division remains difficult to evaluate in clinical tumours. Here, we demonstrate that faster division on the tumour periphery leaves characteristic genetic patterns, which become evident when a phylogenetic tree is reconstructed from spatially sampled cells. Namely, rapidly dividing peripheral lineages branch more extensively and acquire more mutations than slower-dividing centre lineages. We develop a Bayesian state-dependent evolutionary phylodynamic model (SDevo) that quantifies these patterns to infer the differential division rates between peripheral and central cells. We demonstrate that this approach accurately infers spatially varying birth rates of simulated tumours across a range of growth conditions and sampling strategies. We then show that SDevo outperforms state-of-the-art, non-cancer multi-state phylodynamic methods that ignore differential sequence evolution. Finally, we apply SDevo to single-time-point, multi-region sequencing data from clinical hepatocellular carcinomas and find evidence of a three- to six-times-higher division rate on the tumour edge. With the increasing availability of high-resolution, multi-region sequencing, we anticipate that SDevo will be useful in interrogating spatial growth restrictions and could be extended to model non-spatial factors that influence tumour progression.

## Abstract (web summary)

A Bayesian state-dependent evolutionary phylodynamic model (SDevo) quantifies the difference in division rates between cells at the periphery and interior of a tumour. In simulations and using clinical hepatocellular carcinoma data, the authors use SDevo to interrogate spatial patterns in tumour growth.

## Main

Tumours develop and progress via an evolutionary and ecological process wherein cellular sub-populations expand and diversify. Over the course of tumour development, tumour cells acquire genetic mutations and new phenotypes that potentially help them compete for resources and adapt for success in their microenvironment. Understanding this process is critical to predicting clinically important events such as if, how and when cells metastasize or develop resistance to therapy.

Although tumour cell growth and success are often attributed to genetic and epigenetic aberrations, an additional important determinant of cell growth is physical location within the tumour. Position governs access to oxygen, nutrients, pro-growth signalling from the stroma, pH, cell–cell interactions and degree of immune exposure, all of which can affect cellular proliferation^1–7^. Taken together, these effects may combine to create an environment in which cells on the boundary of a tumour have higher growth rates compared to those in the centre (that is, ‘boundary-driven growth’).

Cancer biologists have long been interested in boundary-driven growth because it changes the evolutionary processes and genetic signatures of tumour progression. The evolutionary impact of boundary-driven growth has been explored via evolutionary theory^8,9^, microbial experiments^10–12^, and decades of cancer computational and mathematical models^1–4,13–18^. Such investigations have revealed that boundary-driven growth blunts the efficacy of natural selection in selecting for beneficial (that is, driver) mutations and purging slower growing (but potentially drug-resistant) lineages^19^. Boundary-driven growth should also enhance the effectiveness of adaptive therapy^20,21^ and cell–cell competition in the tumour interior. Further, such growth patterns should distort our expectations for the neutral variant allele frequency (VAF) spectrum^22^, which has been used as a null model for identifying signatures of natural selection^23^, and it has been qualitatively suggested in tumour simulation studies that boundary-driven growth could be misinterpreted as selection on tumour trees^17^. Therefore, establishing and incorporating these null expectations and models for boundary-driven tumour growth is essential in the context of the increasing interest in applying evolutionary theory to clinical disease, for example, in designing adaptive therapy^24^, identifying driver events^25,26^ or estimating timings of metastases^16,27^.

An extensive history of clinical and experimental observations supports the importance of boundary-driven growth in tumour populations. These observations date back to the pioneering work of Thomlinson and Gray, which first identified necrotic structures with surrounding boundaries of growing cells from histological sections^28^, and subsequent cell staining approaches that found markers of cell division cluster preferentially on the tumour periphery^29,30^. Similar patterns have been noted in cultured tumour spheroids^31,32^ and organoids^33,34^. Since then, analysis of both clinical samples—via immunohistochemistry^35,36^, spatial transcriptomics^37–39^ and genetic analysis^40,41^—and experimental systems, such as fluorescentlytracked xenografts^7,42–44^, have further supported spatial heterogeneity and preferential expansion on the tumour periphery in some tumours.

However, more recent studies have hinted at more complex modes of clinical tumour growth. One^41^ found that many colorectal tumours showed genetic patterns not consistent with boundary-driven growth, and a recent genetic analysis of renal cell carcinomas found the most recent common ancestors of metastatic lineages in the resected tumour interiors as opposed to the tumour boundaries^45^. Additionally, experimental evidence suggests that although centre-bound cells may experience oxygen and nutrient deprivation, hypoxia-related signalling can be linked to stem-cell-like tumour phenotypes with increased survival and chemotherapy resistance^46,47^. These observations highlight that higher proliferation on the tumour edge is not necessarily synonymous with long-term lineage survival and progression^48^.

A primary challenge in reconciling these conflicting observations is that clinical sequencing often captures only a limited snapshot of tumour diversity and growth. However, this sampled tumour diversity still offers a window into past population dynamics via phylogenetic and phylodynamic tools. Phylogenetic approaches, which reconstruct how cells within a tumour are related, have already proved useful in interrogating cancer evolution—for example, in determining the relative ordering of driver mutations^49–51^, detecting parallel evolution of gene hits within a tumour^52,53^ and resolving whether metastases emerge early or late in tumour development^54,55^. In contrast, phylodynamic methods, which link shapes of phylogenetic trees to underlying population dynamics, have only rarely been used in cancer genomics^56^, despite widespread application in other fields^57,58^.

Although phylodynamic approaches have high potential impact in cancer clinical settings, they are generally not adapted to study tumour biology or to incorporate the complexities of cancer’s spatial growth. To bridge this gap, we set out to develop a phylodynamic model suited for detecting boundary-driven growth in tumours. First, we quantify characteristic branching and genetic patterns in tumour trees simulated under boundary-driven growth, and demonstrate that these patterns correspond to cellular lineages spending different amounts of time on the faster-growing tumour edge versus in the tumour centre. To fully exploit these patterns for inference, we develop a novel phylodynamic tool based on the multi-type birth–death process^59–61^, in which cells have different birth and death rates on the tumour edge and centre, and lineages can transition between states as the tumour grows. Crucially, we introduce an extension that links cell birth and mutation, and therefore incorporates rates of sequence evolution that depend on each cell lineage’s inferred history of spatial locations (that is, spatial states). We provide this state-dependent evolution model (SDevo) as a package in the popular open-source Bayesian software BEAST 2 (ref. ^62^). We show that SDevo substantially improves our ability to infer boundary-driven growth dynamics in simulated tumours compared to non-cancer multi-type birth–death models, and validate this approach across a broad array of biological and sampling conditions, including those encompassing selection for driver mutations, three-dimensional (3D) growth and clinical sampling strategies. Finally, we apply SDevo to spatially resolved multi-region sequencing data from hepatocellular carcinomas (HCCs)^40^ and estimate that cells on the tumour boundary may have birth rates up to three to six times faster than those in the interior. More broadly, SDevo is a general tool for quantifying growth processes linked to any discrete state, and future investigations will expand beyond boundary-driven growth.

## Results

### Boundary-driven growth creates distinct tree structures

**Fig. 1:**
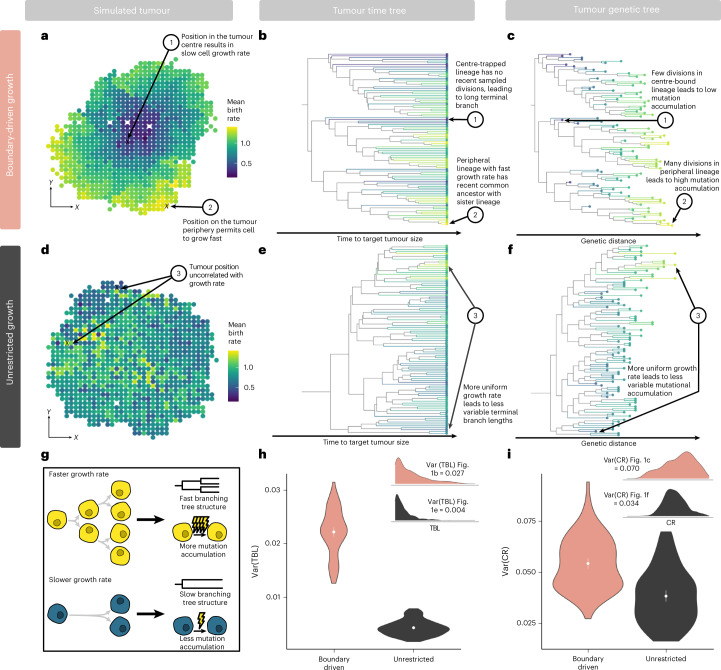
Boundary-driven growth causes characteristic tree patterns associated with asymmetrical division. **a**,**d**, Representative simulated tumours (*α* = 0.004) showing variation in mean birth rate in 2D tumour space under boundary-driven growth via neighbourhood-based spatial constraints (**a**) and unrestricted growth (**d**). Brighter colours represent higher birth rate (total number of divisions in cell lineage/simulation time) throughout all panels. **b**, Time tree of the representative boundary-driven growth tumour (subsampled to 100 cells for visualization) shows high variation in branching rates, leading to long terminal branches of centre-trapped lineages. **c**, Genetic tree of the representative boundary-driven tumour (subsampled to the same 100 cells) shows ladder-like patterns due to mutation being tied to cell division. **e**,**f**, Time (**e**) and genetic trees (**f**) for the representative tumour under unrestricted growth (100 tips visualized) reveal less variation in branching rates and genetic distance. **g**, Cartoon schematic of the two signals of boundary-driven growth in trees left by asymmetric birth rates: variation in branching rates and variation in the number of mutations. **h**,**i**, Variance in terminal branch length (Var(TBL)); **h**) and clock rate (Var(CR)); **i**) in tumours under boundary-driven growth and unrestricted growth trees built from all extant tumour cells. Violin plots summarize statistics across 100 simulated tumours. Means (points) with 95% confidence intervals (error bars) are also indicated. Insets show distributions of TBL and CR signals for tumours plotted in **a** and **b**.

**Extended Data Fig. 1:**
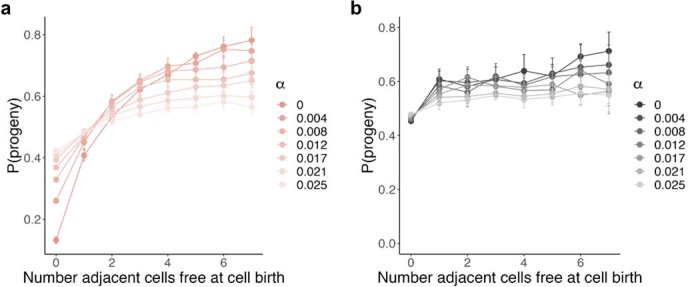
Cellular density creates fitness differences in expanding lattice based simulations. A. Fitness, here approximated by the probability a cell has a daughter cell in the population (P(progeny)) versus the number of adjacent free cells at birth under boundary-driven growth. Spatial impacts on cell fitness are relaxed with increasing cell death rate α (color tint). B. Under unrestricted growth, most cells are born into a dense neighborhood (free cells = 0), but fitness is not impacted by spatial location. For both plots, means and standard error bars are summarized across n=10 simulated tumors per death rate.

In order to characterize signatures of boundary-driven growth in tumour trees, we simulate spatially constrained growth via a cellular agent-based model in a two-dimensional (2D) lattice, following a rich literature of studying cancer dynamics via Eden models^13,17,63,64^. Simulated tumours grow from single cells over discrete time steps and gain mutations at cell division. Under spatially constrained boundary-driven growth, a cell can only divide if there is an empty lattice spot in its Moore (eight-cell) neighbourhood, effectively tying its fitness to neighbourhood density (Extended Data Fig. 1). Therefore, extant lineages closer to the tumour periphery have progressively higher mean birth rates than those in the centre (Fig. 1a). For comparison, we simulated a non-spatially constrained unrestricted growth model (Fig. 1d), in which all cells can divide regardless of density and push their neighbours to create space.

We first investigated how such growth processes affect the shape and structure of cancer phylogenetic trees to identify detectable tree signals of boundary-driven growth. We considered two types of tree representation: (1) time trees (Fig. 1b,e), where the branch lengths are in units of simulation time; and (2) genetic trees (Fig. 1c,f), where the branch lengths are in units of number of mutations. We first compared the time tree of a tumour simulated under boundary-driven growth (Fig. 1b) with one simulated with no spatial restrictions (Fig. 1e). In the boundary-driven growth tree, we observed certain leaves (cells) with long terminal branches (that is, cell 1) and other leaves with much shorter terminal branches (that is, cell 2). These differential terminal branch lengths directly correspond to both mean lineage birth rate and spatial position within the tumour. Intuitively, lineages trapped in dense centre neighbourhoods (that is, cell 1; Fig. 1a,b) divide slowly and therefore exhibit longer times since diverging from another sampled cell. Conversely, lineages at the tumour boundary (that is, cell 2) divide rapidly, and are therefore more likely to be recently related to another sampled cell. We quantified terminal branch lengths in the simulated tumour time trees and found that the asymmetries in birth rates due to spatial constraints result in an overall higher variance in terminal branch lengths under boundary-driven growth than under unrestricted growth (Fig. 1h).

In Fig. 1c,f, we reconstruct the genetic trees from the same boundary-driven and unrestricted tumour simulations. From this representation of the tumour trees, we observe that if mutation is linked to cellular division, then asymmetries in birth rates across tumour space logically correspond to varying rates of sequence evolution (Fig. 1g). This leads to repeated ladder-like patterns of genetic divergence that arise across multiple subclades of the boundary-driven growth tree in which fast-dividing cells on the tumour boundary accumulate more mutations than those in the interior (Fig. 1c). These patterns are not observed in the unrestricted growth tree (Fig. 1f). We quantified these patterns by measuring variance in mean clock rate (defined by total lineage mutations/simulation time) from extant cells in each simulation and demonstrate that clock rate is more variable across trees derived from boundary-driven growth than in trees simulated under the unrestricted growth model (Fig. 1i).

### Two-state birth–death process models boundary-driven growth

**Fig. 2:**
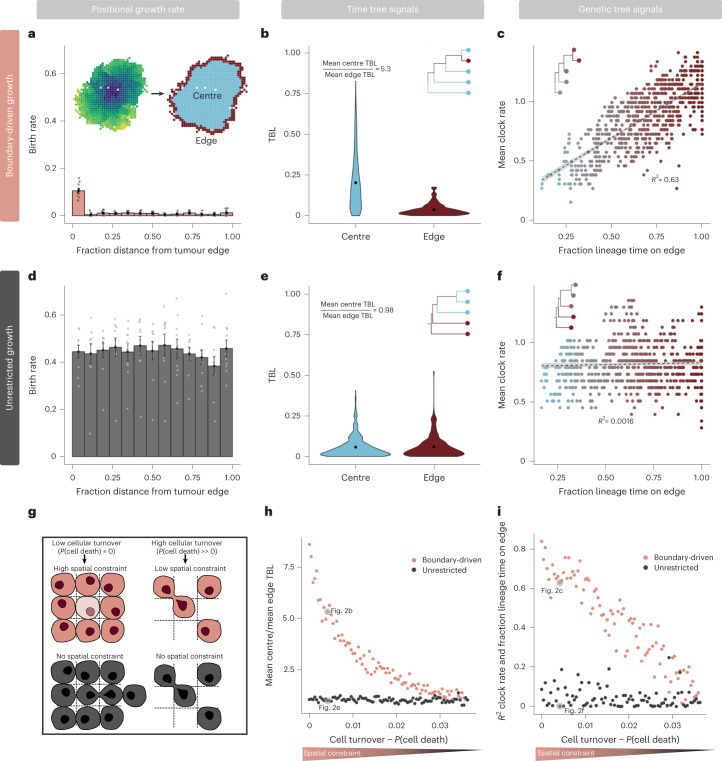
Asymmetries in cell birth rate and signals of boundary-driven growth in trees can be modelled by two-state dynamics. **a**, Histogram of instantaneous cell birth rate as a function of distance from the tumour edge (normalized by maximum distance). Rates are averages over ten simulations under boundary-driven growth (*α* = 0.004) with standard error bars. Points represent individual simulation means. **b**, Distributions of normalized terminal branch lengths (TBL) in a representative tumour under boundary-driven growth (Fig. 1a) categorized by leaf edge or centre state. **c**, Mean clock rate (total number of mutations/time) of cells in the example boundary-driven tumour versus the fraction of time a cell lineage spends on the tumour edge. Colour gradient spans mostly centre-associated lineages in blue to mostly edge-associated lineages in maroon. Dashed line is *y* = *x*. **d**, Histogram of instantaneous cell birth rate versus binned distance from tumour edge in unrestricted growth simulations (average over ten simulations with standard error bars, *α* = 0.004). **e**, Distributions of terminal branch lengths for edge and centre leaves in the representative unrestricted tumour (Fig. 1d). **f**, Average lineage clock rates versus the fraction of time a lineage spends on the tumour edge in the example unrestricted tumour. Insets show representative phylogenetic patterns observed in time (**b** and **e**) and genetic (**c** and **f**) trees. **g**, Schematic comparing simulated spatial constraints under boundary-driven growth (coral) and unrestricted growth (black). **h**, Ratios of centre-to-edge mean terminal branch lengths across simulations with decreasing spatial constraints (as modulated by cell death rate) under either boundary-driven or unrestricted growth modes. **i**, Correlations (measured via *R*^2^) between the fraction of the lineage time spent on the edge and mean clock rate across the same range of spatial constraints and growth modes.

As tree structures differ between tumours simulated under boundary-driven and unrestricted spatial constraints, we sought a phylodynamic approach that could differentiate between these two growth modes. One such model is the multi-type birth–death model^59–61^, which ties differential rates of birth, death and sampling of lineages to multiple discrete states. In our simulation studies, we observe that boundary-driven growth can be effectively simplified into two states. We find that the instantaneous cell birth rate under boundary-driven growth is elevated only in cells immediately adjacent to the tumour edge, but is uniformly low in all cells in the interior (Fig. 2a). We can further decompose the tree patterns observed in Fig. 1 into edge and centre-linked dynamics. As shown in the representative tumour from Fig. 1a, all edge-associated cells have short terminal branch lengths. Most of the variation in terminal branch length can be attributed to cells in the centre, and the mean terminal branch length of cells in the centre is more than five times that of cells on the tumour edge (Fig. 2b). If we trace the lineages of extant cells back to the root, the fraction of time cell lineages spend on the edge is highly correlated with the variation in mean clock rate observed in Fig. 1 (Fig. 2c; *R*^2^ = 0.63). In other words, the most mutated cells have spent the majority of their lineage history on the tumour edge. Under unrestricted growth (Fig. 2d), we observed no difference between edge and centre terminal branch lengths (Fig. 2e; ratio of centre-to-edge mean terminal branch lengths = 0.98), and lineage time spent in the edge state is not correlated to clock rate (Fig. 2f; *R*^2^ = 0.0016).

To investigate the robustness of these patterns, we next simulated tumours under a wide range of cell turnover rates. Under boundary-driven growth, increasing cell turnover decreases spatial constraints and therefore lessens the growth advantage between edge and centre states (Extended Data Fig. 1 and Fig. 2g). We measured the ratio of mean centre-to-edge terminal branch lengths as in Fig. 2b,e across these different effect sizes and found that this ratio is a consistent indicator of boundary-driven growth that decreases as spatial constraints are relaxed (Fig. 2h). The correlation between fraction of lineage time spent on the edge and mean clock rate is also specific to the boundary-driven growth model and sensitive to effect size (Fig. 2i). Therefore, we conclude that the patterns left by boundary-driven growth can be effectively approximated by a two-state birth–death model.

### Phylodynamic models detect signals of spatial constraints

**Fig. 3:**
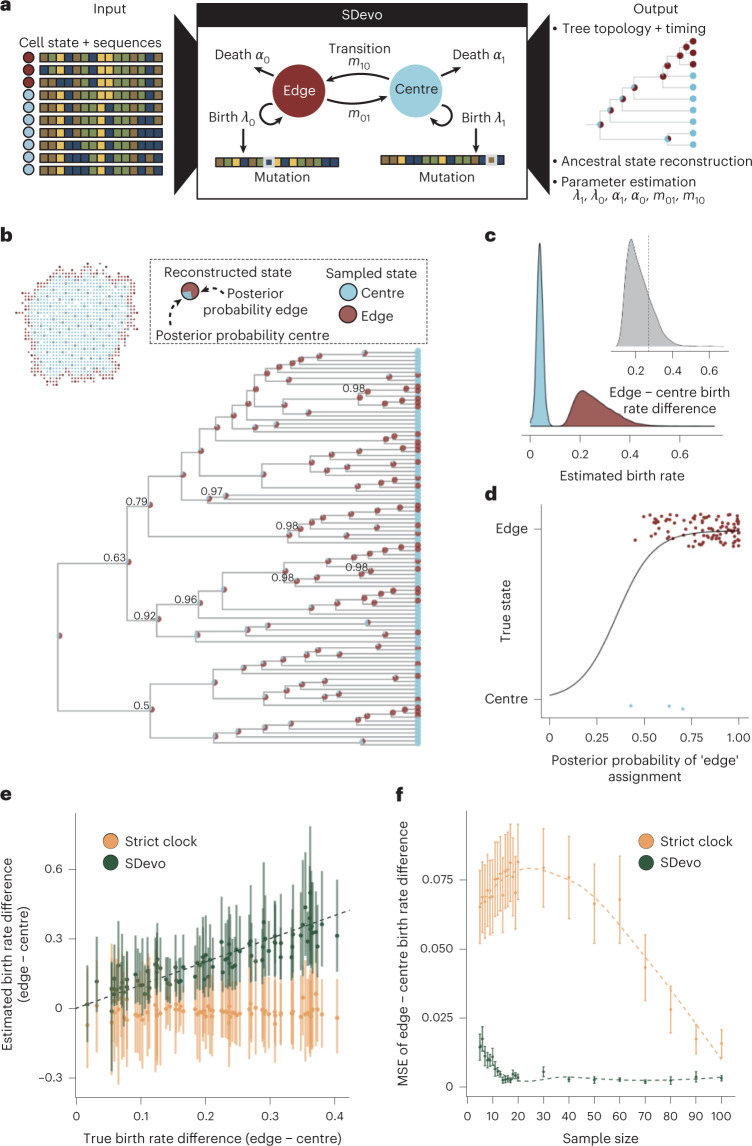
SDevo estimates boundary-driven growth in simulated tumours. **a**, SDevo model schematic. Using input sequences and states (edge in maroon versus centre in blue) of sampled tumour cells, SDevo reconstructs a tree with ancestral states (state probabilities represented by node pie charts) and estimates model parameters. SDevo links state-dependent clock rates to birth rates. **b**, Reconstructed time tree estimated by SDevo on an example simulated tumour (*α* = 0.012). At each internal node, the posterior probabilities for ancestral edge or centre states are shown as a pie chart. Clade posterior support is indicated if less than 99%. The inset shows the sampling scheme for the tumour. **c**, Marginal posterior distributions of estimated edge and centre birth rates, which are summarized by birth rate differences between edge and centre cells (inset, dashed line indicates the true difference). **d**, Posterior probabilities of ancestral state reconstructions versus true state assignments. **e**, SDevo (green) estimates of birth rate differences between edge and centre samples across a variety of true per-day birth rate differences (*α* varies between 0 and 0.036, *n* = 50) compared with estimates under a strict clock (gold). Points and bars represent mean and 95% HPD intervals, respectively. Dashed line is *y* = *x*. **f**, Mean squared error (MSE) of estimated birth rate differences in simulated tumours (*α* varies between 0 and 0.036) versus input number of cells sampled per tumour for SDevo (green) and strict clock sequence evolution (gold) models. Error bars represent the standard error of MSE and are summarized across 17 simulations per sample size.

**Extended Data Fig. 2:**
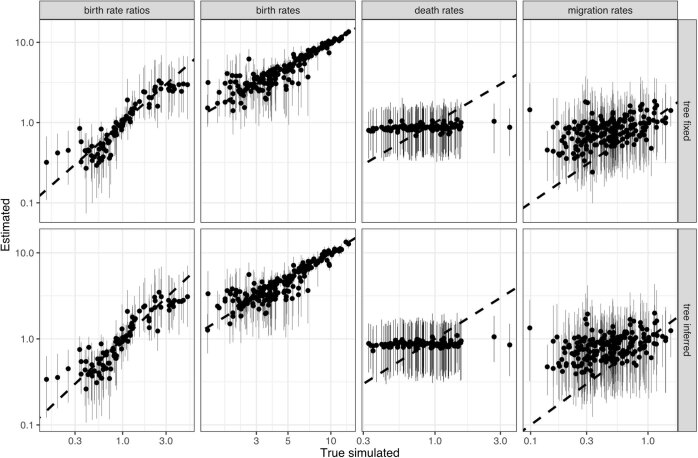
Simulation study to validate SDevo implementation. Birth, death, and transition rates, and ratios of state-dependent birth rates estimated by SDevo versus true population parameters of phylogenetic trees simulated under a two-state birth-death model (see Methods). Medians (points) and 95% HPD intervals (bars) of estimated values are plotted for each parameter (columns) while either fixing or jointly inferring the tree topology (rows). Trees were simulated under a birth-death with migration model with rates drawn from log-normal distributions for birth (mean=5, S=0.5), death (mean=1, S=0.5), and migration (mean=0.5, S=0.5). The sampling proportions were also drawn from a log-normal distribution (mean=0.15, S=0.5). This process leads to trees with between 32 and 72 tips.

Two-state birth–death models incorporate how lineages divide, die, change states and are sampled. In this class of models, birth events correspond to observed branching events on the tree, and the rate of these branching events depends on an underlying type or state. Although existing phylodynamic models, such as BDMM^61,65^ and BiSSE^59^, permit asymmetrical division rates based on state, they do not link birth and mutation. Therefore, although they are well-positioned to infer faster birth rates from branching structures, they cannot learn from differential rates of genetic divergence, a key hallmark of boundary-driven growth we observed in simulations. Additionally, branching patterns are prone to artificial inflation if more cells from a particular state are sampled in a clustered manner^66^. Thus, existing models both do not incorporate all potential signals (that is, clock rate differences) and, importantly, may be biased by sampling procedures in clinical tumour biopsies. To address these shortcomings, we introduce the SDevo model to directly tie state-dependent birth rates to clock rates. This enables the model to combine information, both from mutation and branching patterns that arise from boundary-driven growth (Fig. 3a). SDevo uses genetic sequences sampled from distinct spatial locations, alongside the label of the cell state (here, centre and edge). Using Markov chain Monte Carlo sampling, we explore the posterior distributions of phylogenetic trees jointly with the parameters of SDevo (Extended Data Fig. 2). Inferred trees are time trees, which encompass the order and timing of cellular divergence events and include inferred internal node states, representing the location of unsampled ancestral cells. Model parameters include state-dependent birth and death rates, and the rate at which cells transition between states.

**Extended Data Fig. 3:**
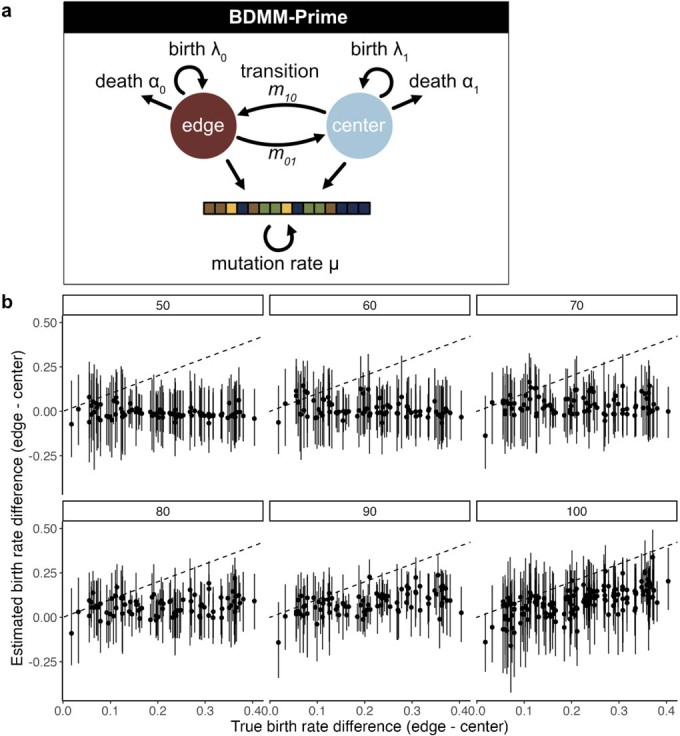
Multi-state diversification model without state-dependent clocks does not sensitively detect birth rate differences in simulated tumors. A. Schematic of BDMM-Prime, which does not link state-dependent effects on division to sequence evolution. B. True versus estimated means (points) and 95% HPD intervals (bars) of per day birth rate differences between the edge and center of simulated boundary-driven tumors over a range of sample sizes (n = 50, 60, 70, 80, 90, 100, see headers). Dashed line is y = x.

We first demonstrate the utility of SDevo on simulated tumours undergoing boundary-driven growth. From the genetic sequences and labelled cell states for sampled cells isolated at a simulated tumour endpoint (Fig. 3b inset), SDevo reconstructs the most likely relationship among sampled cells and the time at which those cells diverged (Fig. 3b). The birth rates for edge and centre-associated cells are inferred from the branching and mutational structure of sampled extant cells (leaves on the tree), permitting quantification of overall birth rate differences between the two spatial compartments (Fig. 3c). SDevo correctly identifies that boundary-linked cells have a higher birth rate than centre-linked cells (mean edge birth rate advantage = 0.22, 95% highest posterior density (HPD) interval = 0.12–0.35, true value = 0.27 in the representative simulation). SDevo additionally reconstructs the probability of each spatial state (centre versus edge) for the ancestors of the sampled population (plotted as pie charts on the internal nodes of Fig. 3b). These reconstructions suggest that the majority of ancestors divided on the tumour edge, consistent with the findings of ref. ^41^ and our expectations of boundary-driven growth. We further quantify confidence in its ancestral reconstructions: ancestral cells with the highest posterior probability of existing on the tumour edge were indeed likely to have divided there (Fig. 3d). On the other hand, cells with more uncertain ancestral reconstructions are less likely to have been on the tumour edge at division (Fig. 3d). Finally, we applied SDevo to tumours simulated under a range of spatial constraints (Methods). We find that at a moderate sample size (*n* = 50), SDevo is able to accurately quantify birth rate differences, whereas a two-state birth–death model without a state-dependent clock (estimated using BDMM-Prime and a strict clock model) fails (Fig. 3e and Extended Data Fig. 3). We further observed that SDevo remains accurate for as few as ten samples, whereas a strict clock model requires >100 samples to reach close to the same accuracy (Fig. 3f).

### SDevo is robust to variation in sampling and growth modes

**Fig. 4:**
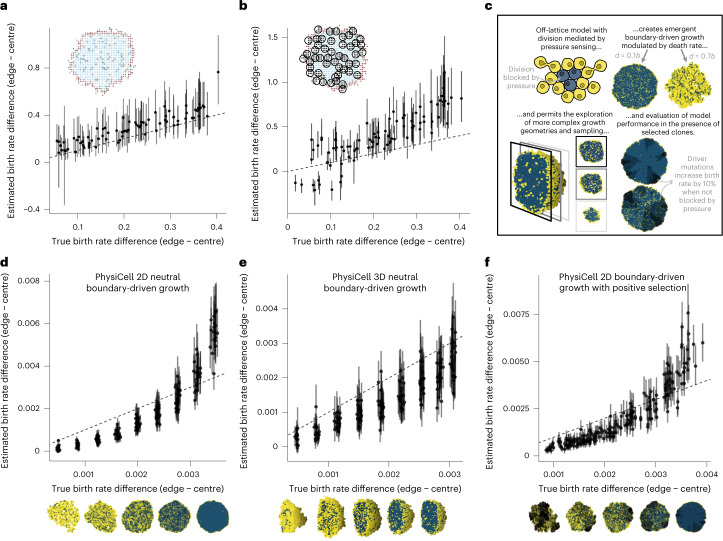
SDevo is robust to a variety of sampling approaches and growth modes. **a**, Mean and 95% HPD interval of estimated versus true edge − centre per-day birth rate differences with random sampling (*n* = 100 cells per tumour). **b**, Mean and 95% HPD interval of SDevo estimates of birth rate differences between edge and centre when sequences are constructed from variants above 30% frequency in a simulated punch biopsy (inset, *n* = 50 punches per tumour). **c**, Schematic of continuous-space tumour growth simulation governed by biomechanics using PhysiCell^70^. Only cells under low physical mechanical pressure from their neighbours (visualized in yellow as opposed to blue) can divide, generating boundary-driven growth. **d**–**f**, SDevo recovers growth rate differences generated through variable death rates under neutral 2D growth (**d**), neutral 3D growth (**e**) and 2D growth in the presence of strong driver mutations (**f**; *μ*_driver_ = 0.01, *n* = 100 cells per tumour, driver fitness advantage = 10%; Methods). Tumour snapshots below the *x* axes show representative examples of growth dynamics under variable death rates (right to left: *d* = 0, 0.2, 0.4, 0.6, 0.8) and resultant pressure. In the case of the tumours under selection shown in **f**, darker colours represent cells with driver mutations. Points and bars represent means and 95% HPD intervals, respectively, for all plots.

**Extended Data Fig. 4:**
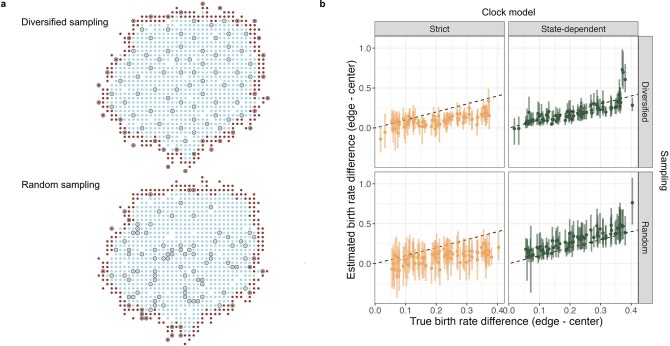
SDevo improves birth rate estimation with more variable (random) sampling over a strict clock model. A. Example 2D tumors under either diversified or random sampling schemes. Cells are colored by edge (maroon) or center (blue). Grey-highlighted cells are sampled. Diversified sampling maximizes the physical distance between sampled cells. B. Estimated means (points) and 95% HPD intervals (bars) of per day birth rate differences between the edge and center of simulated boundary-driven tumors based on n = 100 sampled cells versus true state-dependent effects (α varies between 0 and 0.036). We compare SDevo (green) with a strict clock model (gold) for either diversified or random sampling (rows).

To evaluate SDevo’s strengths and limitations in clinical tumours, we sought to validate that SDevo detects boundary-driven growth under various sampling strategies. Whereas in the initial simulation studies we maximized the distance between sampled cells (that is, diversified sampling), we also implemented a random sampling scheme as might be present in single-cell studies (Extended Data Fig. 4a). Under random sampling, cells sampled close together provide minimal additional genetic information but may create spurious signatures of rapid branching. Despite this, SDevo successfully estimates edge-driven birth advantages from randomly sampled cells (Extended Data Fig. 4b). In contrast, even with a large number of cells sampled (*n* = 100), the strict clock multi-type birth–death model often fails to detect the same birth rate differences (Extended Data Fig. 4b). We also assessed SDevo’s robustness to punch biopsy sampling, in which a population of nearby cells are captured. We biopsy-sampled our simulated tumours, and only called mutations exceeding a 0.3 cellular fraction threshold within a punch (Methods). We find that while punch-style sampling adds more random error due to variation in sampled diversity, especially in tumours with high turnover rates, SDevo largely still detects state-dependent birth rate effects (Fig. 4b).

**Extended Data Fig. 5:**
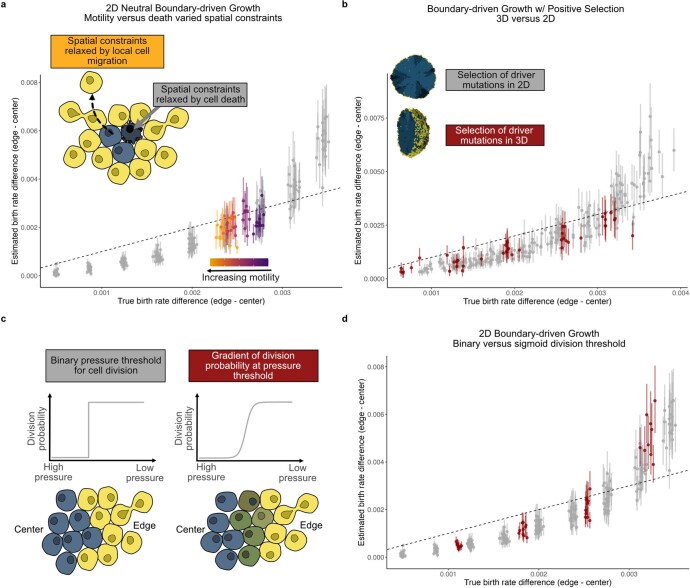
Investigation of extended modes of spatial tumor growth in PhysiCell simulations. A. Mean (points) and 95% HPD intervals (bars) of birth rate differences estimated by SDevo when spatial constraints are relaxed by increasing cell motility (purple to orange gradient) compared to when spatial constraints are relaxed by cell death (grey). N = 100 per tumor for all simulations. The x-axis is the effective true birth rate difference in both scenarios. B. True versus SDevo-estimated birth rate differences in simulations with both boundary-driven growth and positive selection of driver mutations (n = 100 sampled cells per tumor). We compare simulations in 2D (grey) and 3D (red). C. Schematic of simulated relationships between cell pressure and division probability for either a binary (left) or sigmoidal (right) gradient in PhysiCell simulations. D. True versus estimated birth rate differences of simulated tumors (n = 100 sampled cells per tumor) with either a binary (grey) or sigmoidal (red) pressure threshold. For A, B, and D. points and bars represent the mean and 95% HPD interval of each estimate.

**Extended Data Fig. 6:**
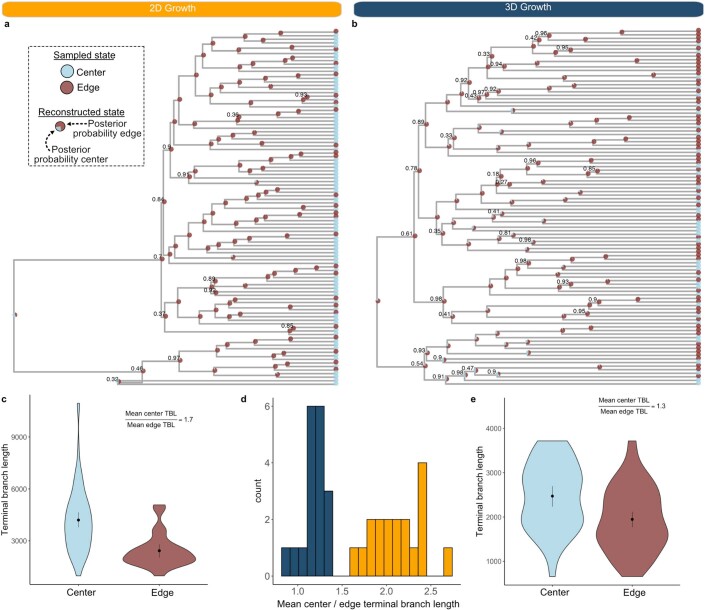
Complex growth and sampling in 3D tumors lead to more variable branching patterns. A. Example inferred phylogeny of 2D PhysiCell tumor with reconstructed ancestral edge and center states (d = 0.1). Node pie charts represent posterior support for each state. 100 cells were sampled to maximize the distance between cells (diversified sampling). B. Example inferred phylogeny of 3D PhysiCell tumor with reconstructed ancestral edge and center states (d = 0.1). Cells were sampled to maximize distance in 2D space across z-slices of the simulated tumor as described in Methods. For both trees, posterior node support is indicated if less than 99%. C, E. Comparisons of inferred terminal branch lengths between cells sampled on the edge and center of the example 2D and 3D tumors. Points and error bars represent means and 95% confidence intervals for n = 75 center and n = 25 edge terminal branches. D. Distribution of the relative ratio of center-to-edge mean terminal branch lengths across multiple simulations with equivalent spatial constraints. Asymmetric branching between edge and center states is observed more often in 2D (gold) than 3D (navy) tumors. Points and error bars represent means and 95% confidence intervals for n = 38 center and n = 62 edge terminal branches, respectively.

Next, we assessed SDevo’s robustness to more complex growth models by exploring an off-lattice model, a more flexible class of spatial models also employed to study tumour evolutionary dynamics^67–69^. We simulated under a continuous space model of tumour growth implemented using the agent-based cellular engine PhysiCell^70^. To mimic boundary-driven conditions, we linked the cellular division probability to mechanical pressure—cells crowded by their neighbours could not divide (Methods and Fig. 4c). As in the lattice-based simulations, higher cell turnover relaxes mechanical pressure, modulating spatial constraints. We first verified that SDevo continued to identify birth rate differences in these more complex simulations. We simulated 2D neutral growth and found that SDevo sensitively detects an elevated birth rate at the tumour edge, even when birth rate differences were minimal (Fig. 4d). However, SDevo slightly underestimates the birth rate differences at high death rates (that is, low birth rate differences). We also confirmed that SDevo was robust to spatial division rate heterogeneity induced by increasing cell migration, as opposed to cell death (Extended Data Fig. 5a), and to a sigmoidal pressure threshold for cell proliferation (Extended Data Fig. 5c,d). We next simulated tumours grown in 3D and sampled across multiple *z*-slices, mimicking clinical sampling approaches. We determined that SDevo accurately reconstructs birth rate differences, albeit with wider posterior intervals (Fig. 4e). We note that trees reconstructed from the 3D simulations tend to deviate more from expected edge-biased branching patterns than those from the 2D simulations (Extended Data Fig. 6), reflecting more complicated growth dynamics and potential obfuscation via the sampling scheme. These observations further highlight the necessity of incorporating both branching and clock rate patterns to quantify boundary-driven growth in clinical scenarios.

Finally, we tested the extent to which SDevo detects boundary-driven growth dynamics when both spatially determined and cell-intrinsic fitness differences influence growth, as the action of strong positive selection has been previously shown to distort the shape of tumour phylogenetic trees^17,40,41^. We find that SDevo continues to detect differences in birth rates between centre and periphery-associated cells even in the presence of strong selection (Fig. 4f, Extended Data Fig. 5b and Methods). Notably, even as lineages with driver mutations expand, these cells are still subject to spatial constraints. As a result, similar patterns of branching and clock rate differences between centre and periphery-associated cells re-emerge. However, we anticipate that if cell death is sufficiently high, a driver mutation could lead to rapid expansion of a centre-bound lineage and mask signals of boundary-driven growth.

### Boundary-driven growth in HCCs

**Fig. 5:**
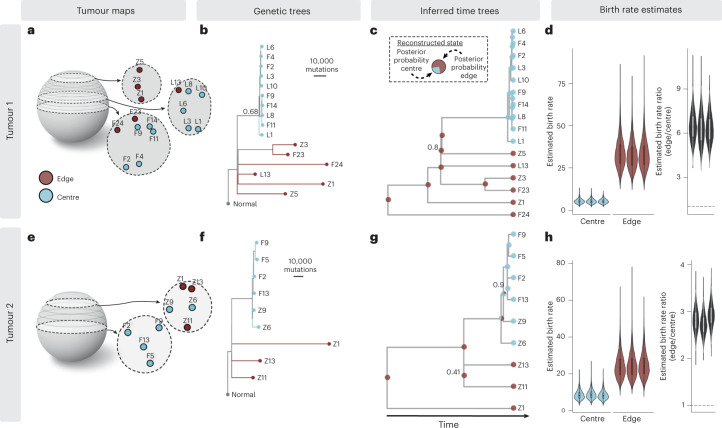
Quantification of boundary-driven growth in HCCs. **a**, Multi-region 3D sampling map for Tumour 1 adapted from ref. ^40^. Sampling locations are marked and labelled in *z*-slices, and centre and edge classifications (taken from the original study) are shown in blue and maroon, respectively. **b**, Maximum likelihood genetic tree reconstructed from all variable sites. Node confidence is labelled if less than 99%. **c**, Tumour 1 phylogeny reconstructed from pseudo-sequences of one subset of variable sites. Tip colours indicate sampled punch state and pie charts on internal tree nodes represent posterior probabilities of ancestral state reconstructions. Clade supports are indicated at nodes if less than 99%. **d**, Marginal posterior distributions for edge (maroon) and centre (blue) birth rates across three independent subsamples of variable sites, and estimated edge-to-centre birth ratio (mean 6.35). Posterior distributions summarize across three independent BEAST 2 runs using all *n* = 16 punches. Dashed line marks ratio of 1. **e**–**h**, The same plots as in **a** (**e**), **b** (**f**), **c** (**g**) and **d** (**h**) are shown for an additional HCC tumour (Tumour 2, *n* = 9) from the same dataset, estimating a mean 2.83 edge-to-centre birth rate ratio. Tumour schematics are vertically inverted for ease of visualization. Violin plots show distribution density and the boxplots show the interquartile range.

**Extended Data Fig. 7:**
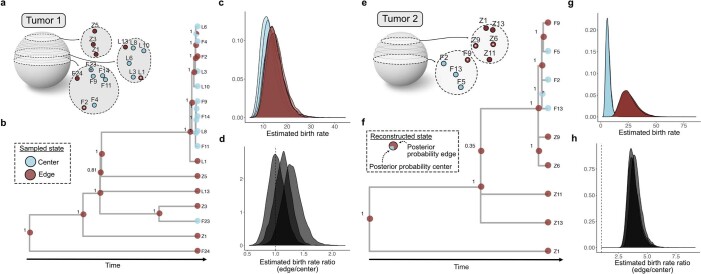
Detection of boundary-driven growth in hepatocellular carcinoma with variation in edge/center state calling. We called an alternative set of states based on a distance of < 10% of each tumor diameter from the edge instead of published edge/center labels. A. Multi-region sampling map for Tumor 1 adapted from Li et al.^40^ with alternative state labels. Asterisk indicates a change in state from published states (Fig. 5A). B. Inferred tumor phylogeny and reconstructed ancestral spatial states for a single SNV subset. Clade posterior supports are indicated at nodes. C. Marginal posterior distributions for edge (maroon) and center (blue) birth rates estimated from the Tumor 1 WGS data across three independent SNV subsets. D. Posterior distribution of edge/center birth rate ratio. Dashed line indicates ratio of 1. We estimate a mean 1.15x higher birth rate on the edge compared to center. E. Multi-region sampling map with alternative states for Tumor reproduced from Li et al.^40^. F. Tumor 2 MCC tree and ancestral edge/center states inferred from the sampled populations. G. Marginal posterior distributions for edge and center estimated birth rates and H. edge/center ratio. We estimate a mean 3.89x higher birth rate on the edge versus center based on the alternative state calls.

**Extended Data Fig. 8:**
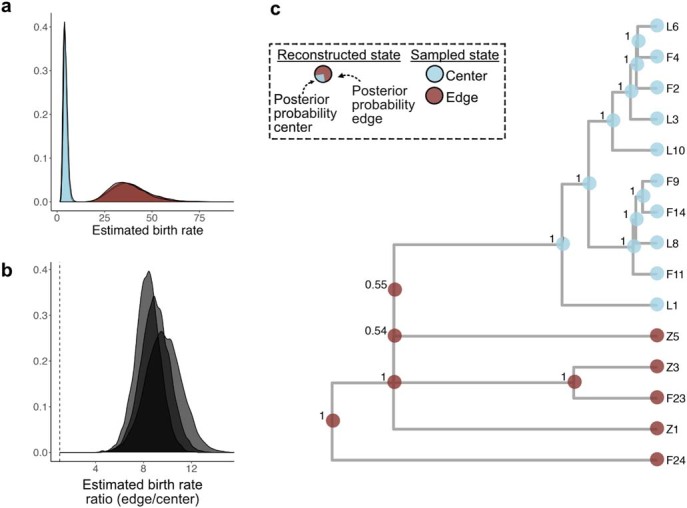
SDevo analysis excluding heterogeneous punch T1L13 also estimates birth rate differences between edge and center. To ensure our results are not driven by an edge-associated sample (T1L13), which potentially contains multiple subclones, we repeated the analysis of Tumor 1 without this punch. A. Marginal posterior distributions or edge (maroon) and center (blue) birth rates estimated from the Tumor 1 WGS data excluding T1L13 inferred for three SNV subsets. B. We estimate a 8.99x higher birth rate on the edge compared to center (95% HPD interval 6.37-11.91x, summarized across the same three independent inferences). C. Inferred MCC tumor phylogeny and reconstructed ancestral spatial states. Posterior clade support is indicated at each node.

**Extended Data Fig. 9:**
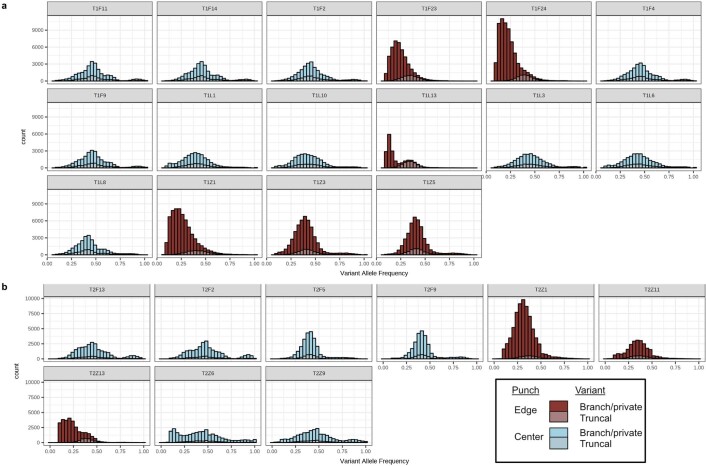
Variant allele frequency (VAF) histograms reveal punches are largely clonal. Variant allele frequencies for all non-truncal (opaque) and truncal (transparent) mutations observed in tumor punches from Tumor 1 (A) and Tumor 2 (B) reveal that punches contain only a single high-frequency clone, with the exception of T1L13. Punches are colored by their edge (maroon) or center (blue) status. State labels correspond to Li et al.^40^, Table S8.

To quantify boundary-driven growth in a clinical tumour setting, we applied SDevo to multi-region sequencing data of two HCC cancers published by ref. ^40^ (Fig. 5). The authors sequenced two HCC tumours from a single patient, carried out 3D spatial micro-biopsy sampling followed by whole-genome sequencing (Fig. 5a,e), and classified punches as ‘edge’ or ‘centre’. The genetic maximum likelihood trees of each tumour (Fig. 5b,f) qualitatively demonstrate an increased genetic divergence at edge punches. To apply SDevo, we created input pseudo-sequences for each punch using three independent 25,000 single nucleotide variant (SNV) random subsets of those identified in the original study. We assumed unidirectional transition from edge to centre, in line with biological expectations of solid tumour growth, to constrain death and transition rate parameter space (Methods). SDevo jointly reconstructed tumour time trees along with the most likely ancestral internal node states. From these results we infer that while most ancestral cells divided on the tumour periphery, some population expansion occurred in the tumour centre. We note that we do not use a predefined outgroup for this analysis, so there are slight differences in rooting for these time trees compared with the genetic maximum likelihood trees. SDevo found strong support for birth rate differences between edge and centre in both tumours (Fig. 5d,h). We estimated that cells on the edge have a mean 6.35× birth rate advantage over centre cells in Tumour 1 (95% HPD interval = 4.53–8.32×) and a mean 2.83× birth rate advantage in Tumour 2 (95% HPD interval = 2.35–3.32×) summarized across all SNV subsets. To assess how sensitive these results were to differences in state classifications or punch heterogeneity, we also called alternative edge/centre states based on a threshold of 10% of the tumour diameter ( ~2 mm and ~1.5 mm for Tumour 1 and Tumour 2, respectively) from the schematic boundary (Extended Data Fig. 7a,e). We found consistent results for Tumour 2, but observed that Tumour 1’s alternative edge/centre classifications showed more variable and reduced support for boundary-driven growth, which was not unexpected given that the alternative states updated the classification of previously centre-assigned punches with less genetic divergence to edge (Extended Data Fig. 7d,h). We further found consistent results when removing a single punch from Tumour 1 (Extended Data Fig. 8), which may have captured multiple subclones (Extended Data Fig. 9).

**Extended Data Fig. 10:**
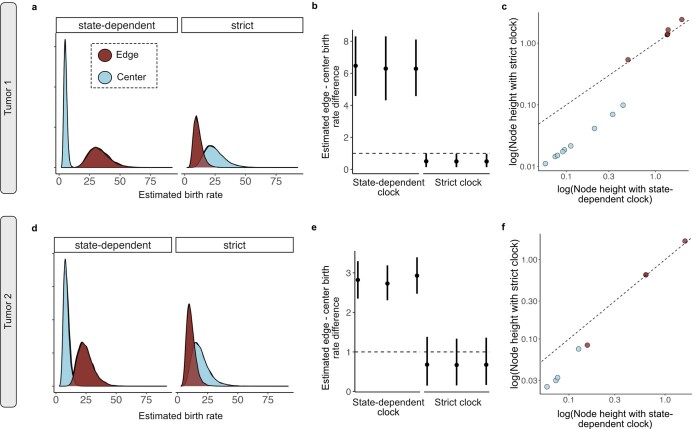
SDevo infers boundary-driven growth in HCC tumors where a strict clock fails and changes inferred node timings. We compared estimates of birth rate differences between edge and center under a state-dependent birth-death model (BDMM-Prime) using both our novel state-linked sequence evolution model or a strict clock (state-independent) sequence evolution model. For Tumor 1 (A), Tumor 2 (D), posteriors of edge and center birth rate estimates for each sequence evolution model are shown in maroon and blue, respectively. Means and 95% HPD intervals (points and bars) for the inferred birth rate ratios between edge and center states for Tumor 1 (B) and Tumor 2 (E). Posteriors are inferred across three independent SNV subsets. Dashed lines indicate ratio of 1. Note, power analyses on simulated tumors (Fig. 3 and Extended Data Fig. 3) suggest that the strict clock model should be under-powered and sensitive to sampling variation at these sample sizes (Tumor 1: n = 16, Tumor 2: n = 9). C. and F. Scatterplots show ancestral node heights inferred under strict clock versus heights inferred by SDevo colored by most probable ancestral state. Nodes are compared based on matching a subset of tips.

Although we inferred a higher birth rate on the edge in these clinical tumours, the branching rate patterns in tumours 1 and 2 qualitatively did not match our expectations from simulations. These branching patterns are potentially influenced by selection, as noted originally in ref. ^40^, or by the non-uniform sampling scheme (Fig. 5a,e). Probably due to these branching patterns, we find a strict clock model, which assumes independence of sequence evolution and cell division, did not detect boundary-driven growth. Instead, it estimated that centre cells have a slightly higher birth rate (Extended Data Fig. 10). We note that the sample sizes of Tumour 1 and Tumour 2 were well below the sample size requirements in simulations to detect boundary-driven growth with a strict clock model (Fig. 3f). In addition, we found that incorporating a state-dependent sequence evolution model changed the estimated internal node timings (Extended Data Fig. 10c,f). Specifically, reconstructed centre-bound nodes were estimated to have occurred more recently under a strict clock than under a state-dependent evolution model in which centre cells would be expected to divide less frequently.

## Discussion

Tumour evolutionary progression is a complex process driven by genetic, epigenetic, environmental and immune factors. Quantitatively disentangling the contribution of spatial factors to tumour growth dynamics is an important component of both reconstructing tumour clinical histories and predicting future growth. Our understanding of spatial drivers of tumour growth has largely been informed by experimental models, as we have had limited ability to assay for these effects in clinical tumours. Here, we introduce SDevo, a new Bayesian phylodynamic model that learns differential cell birth rates of discrete classes (here, tumour periphery or centre-associated). Although SDevo is general in scope and applicability, here we demonstrate that it successfully infers birth rate differences between the tumour edge and centre from multi-region sequencing data. We show that SDevo is relatively robust to sampling choices (that is, punch biopsies and locations) and biological factors (that is, cancer driver mutations and 3D versus 2D growth modes). We further find quantitative evidence for boundary-driven growth in clinically derived HCCs resected at a single time point.

Our assessment of boundary-driven growth in HCCs quantitatively expands the observations of ref. ^40^. The authors originally hypothesized that Tumour 1’s tree structure matched a simulated scenario of boundary-driven growth followed by the expansion of a selected clone in the centre and that Tumour 2’s tree structure matched dominant boundary-driven growth. The authors made these assessments by simulating tumours and comparing the distributions of clones and variant allele frequencies to the sequenced tumours. They further noted that genetic divergence was higher in punches collected from the tumour periphery.

Our study quantifies these patterns by estimating these birth rate differences directly with joint inference of tree topology and sequence evolution. Notably, although small sample sizes, clustered sampling and the hypothesized selection for an internal clone in Tumour 1 may have distorted the branching structure of the trees, SDevo is able to detect past boundary-driven growth from clock rate differences. By explicitly incorporating the mutational process, SDevo leverages data more effectively than models that only learn from state-dependent branching. This approach is particularly important when only a few areas of a tumour are sequenced. These findings, along with previous in silico evidence that selection changes the shapes of tumour trees^17,71^, highlight the importance of employing multiple tree patterns to quantify interacting modes of tumour growth. Although future work should more comprehensively profile how multiple spatial and non-spatial drivers of growth can impact observed tree patterns, our analysis of non-neutral tumours (Fig. 4f and Extended Data Fig. 5b) suggests that SDevo can detect boundary-driven growth in the presence of selection.

Quantifying the impact of spatial restrictions on clinical tumour growth informs how we understand, predict and control cancer evolution. A robust literature has established that boundary-driven growth modulates the efficiency of positive and purifying selection^19,72^, alters overall growth rates^43,73^, and increases the efficacy of adaptive therapy^20–22^. Spatial restrictions also change the expected distribution of genetic variation in solid tumours^13,15,16,72^ and impact how clinically informative biopsies should be collected^74^. Although we find robust evidence for boundary-driven growth in HCCs, its prevalence and strength probably vary by stage of tumour growth and tumour type^18^. For example, increased vascularization, cellular migration, physical anatomical structures or tumours reaching a local carrying capacity could alter spatial growth restrictions. Further applications of SDevo to other tumour cases and types will enable us to explore the nuances of these growth phenomena.

Importantly, the utility of SDevo is not limited to understanding the impact of boundary-driven growth, but in fact can be applied in any instance in which sequenced tumour samples can be classified into discrete, observable states. Immediately, SDevo could be extended to test other proposed tumour growth modes—for example, growth against a solid surface, such as bone in osteosarcoma, along a unidirectional invasive front^75^, or in different glandular compartments^76^. Because tumours can grow under a wide variety of anatomical constraints, integrating system-specific factors can help assign biologically relevant environmental states for the application of SDevo (that is, edge categorization may constitute those cells that have penetrated the basal layer as opposed to those that are most radially extreme). Even more broadly, SDevo could be applied to study the growth impacts of other environmental or cell-intrinsic factors, for instance, immune invasion, hypoxia, metastatic versus primary sites or genetic features, by decomposing complex phenotypes into discrete states.

Phylodynamic approaches such as SDevo have major advantages compared with our current approaches for estimating evolutionary information from tumours, namely approximate Bayesian computation (ABC)^77^ or other approaches that compare simulated and clinical tumours via summary statistics^18^. To be clear, these approaches have yielded extensive insights into tumour evolution, including patterns under boundary-driven growth^14,15,17,41^. However, these approaches are computationally costly, requiring the generation of often tens or hundreds of thousands of simulated tumours, on which one must compute extensive summary statistics. In addition, ABC comes with technical challenges, including the necessary choice (and potential unavailability) of low-dimensional sufficient summary statistics. Although Bayesian phylodynamics comes with its own technical challenges (that is identifiability, sensitivity to model assumptions, choice of priors; see refs. ^78,79^), it does not require tumour simulation. Furthermore, the generality of discrete traits affecting growth dynamics means it is easily adaptable to answer new questions. While both ABC and phylodynamics offer ways to understand clinically derived samples, the full promise of phylodynamics has yet to be widely exploited.

Phylodynamic approaches to understanding tumour evolution offer additional benefits. (1) Used in conjunction with well-calibrated molecular clocks, inferred trees can help estimate the timing of clinically important events, such as the emergence of subclones or metastatic events. While these analyses have been employed in the context of uniform growth rates^56,80,81^, the expansion of tree models to permit differential birth rates could improve timing accuracy. (2) Incorporating differential growth rates across a tree can lead to more accurate tree topologies, as has been demonstrated in influenza evolving in multiple host species^82^. (3) Inferring ancestral states can elucidate population history and tumour evolutionary processes at time points that cannot be clinically sampled. A recent study^45^ analysed the intra-tumour spatial and genetic architecture of renal cancers and concluded cells in the tumour centre are more likely to seed metastasis. However, the study was limited to observing the extant position of these samples, whereas SDevo reconstructs these states at the time of clinical events (that is, divergence of a metastatic clone). These three points suggest more broadly how tumour trees can be leveraged to gain new quantitative insights into tumour evolution, and demonstrate the broad utility of modelling evolutionary processes on trees.

Beyond its application to cancer evolution, SDevo is a novel phylodynamic model with broad usefulness to incorporate state-dependent clock rates into evolutionary inference. While the field of phylogenetics has developed a broad array of clock models, to our knowledge, SDevo represents the first model in which clock rate is linked to birth rate. SDevo could be particularly useful in microbial and viral populations where diversification and mutational accumulation operate on similar timescales, and may be linked to underlying state variables (for example, location). We demonstrated that incorporating clock rate differences, instead of relying solely on tree diversification rates (as in BDMM and other multi-state birth–death models^59,61,65^), can improve inference in cases where sampling may be non-uniform. This may be particularly important when sampling rates vary, for example, countries with variable rates of molecular surveillance for SARS-CoV-2. To facilitate broad application, SDevo is built as a package in the popular Bayesian phylogenetic platform BEAST 2^62^. As with all phylodynamic models, identifiability represents a pervasive concern, but incorporating biological knowledge for determining priors can help constrain the model space. In our analysis of HCCs, we use information about cell transition and death rates to distinguish between multiple parameters that impact trees and estimation in interrelated ways.

Biological complexity within tumours can complicate SDevo’s application and interpretation via spatially or temporally varying selection. First, strong selection can destroy or alter signals of boundary-driven growth^17,40^. For example, a hard bottleneck, as in the cases of surgery or chemotherapy, would probably temporarily destroy signals of boundary-driven growth. Such signals would probably also re-emerge were the tumour to regrow via boundary-driven growth. Second, gain of driver mutations will lead to cell-intrinsic fitness differences that may not correlate with spatial location. Third, disentangling boundary-driven dynamics from other environmental or cell-intrinsic factors could be especially difficult under time-varying selection. For example, angiogenesis could increase resources to centre cells later in tumour growth^83^, and complex cell-to-cell interactions may create frequency dependencies that further complicate observed spatial patterns^21,48,84^. We have shown that SDevo can detect signals of boundary-driven growth even with driver-induced selection, but future work should further probe this robustness.

Although SDevo is a powerful tool, we note several important limitations that require further caution when applying it to data. First, SDevo assumes mutations occur at cell division. If, instead, most mutations emerge due to exogenous processes^85^, birth-driven genetic divergence could be masked. While this might decrease SDevo’s power, exogenous mutational processes distributed evenly across a tumour are unlikely to generate false positive signals of boundary-driven growth. Second, extensive cell mobility could weaken signatures of boundary-driven growth even if boundary-associated cells have birth rate advantages. Third, as we demonstrate in Fig. 3, sample sizes must be sufficient to detect state-dependent effects. We maximize limited sample sizes by choosing priors that are biologically informed (for example, unidirectional state transitions), but larger sample sizes will enable inference with less informative priors. Data sets that meet this requirement are becoming rapidly available, so we anticipate phylodynamic models such as SDevo becoming increasingly powerful.

The expanded application of phylodynamics to cancer sequencing data relies both on developing methods to exploit single-cell sequencing data^86,87^, and understanding the relationship between sequenced multi-region punches and the many single cells that comprise them. As has been noted previously, multi-region sequence trees are not phylogenies^88^, and punch-wide genetic composition does not necessarily capture all cellular genotypes^89^. Although SDevo is fairly robust to our simulated punch-style sampling and we analysed HCC data from small, largely homogeneous punch biopsies, best practices for applying phylodynamic models to trees of deconvoluted clones are an important area for future research.

Applying phylodynamic methods to tumour populations is in its infancy, but new methods that overcome the barriers of working with tumour data will help extend the applicability of these approaches^86,90^. Here, we demonstrate the utility of phylodynamic models in quantifying spatial factors driving cancer progression. As technologies enabling the widespread and high-throughput generation of tumour trees advance^71,91^, we expect adapted phylodynamic approaches such as SDevo to provide a rigorous analytical toolkit for extracting quantitative insights from these data.

## Methods

### Tumour simulations

#### Eden model

An agent-based model was implemented in Python3, which places simulated cells on a 2D lattice. Simulations are initiated with a single cell in the centre of the lattice. At each time step (2 h) cells have a probability of dying (*α*) and a probability of attempting division given survival (*λ*). Under boundary-driven growth, cells only successfully divide if there is an empty lattice spot in their Moore neighbourhood. If multiple neighbouring spaces are available then a cell randomly chooses the location for its daughter cell from open neighbouring spaces. Under unrestricted growth, if a cell attempts division, its daughter cell will occupy an empty lattice spot in the Moore neighbourhood if available, but if not, the cell will still divide and push cells in a random direction to make space. Overlapping cells are pushed in the same direction until a neighbouring lattice spot is available, which the pushed cell will occupy. In both simulations, if a cell divides, each daughter cell can gain mutations with probability *μ* (per division per genome). Mutations are then drawn from a Jukes–Cantor model of sequence evolution and follow an infinite-sites assumption. Therefore, each time a mutation is gained, a site is added to all cells in the simulation. Simulations are stopped when the number of living cells is more than 1,000. The ground truth birth rates are assessed at discrete time points in the simulation by recording the current state of each cell and the proportion of cells that have progeny in the next time step. True birth rates are considered to be the mean across all time steps weighted by the number of cells in each category. This method calculates effective birth and death rates on the edge and centre given the simulated spatial constraints by calculating empirical division rates on the edge and centre of cells through simulated time. Effective spatial constraints in the boundary-driven model were controlled by changing the probability of cell death, where increased cell turnover allows centre-trapped cells to divide more readily (Extended Data Fig. 1). To evaluate the accuracy of parameter estimation, we ran 1,000-cell tumour simulations where the probability of cell death per time step, *α*, varied from 0 to 0.036, the probability of attempting division given survival, *λ*, was 0.04 and the rate of mutation per division was *μ* = 1. Not accounting for spatial constraints, these birth and death parameters translate to an approximate 0.32–0.40 per day probability of division (per cell) and a range of 0–0.35 per day (per cell) probability of death. Although clinical tumours have large variability in rates of proliferation, death and mutation, these parameters fit within this biological range^13,92–94^.

#### Eden tree statistics

Tree statistics in Figs. 1 and 2 were calculated from simulated tumour trees that include all extant cells. Normalized terminal branch lengths were calculated by dividing terminal branch lengths of tumour time trees by total simulation time. Clock rates were calculated by dividing the total number of mutations accumulated in each alive cell by simulation time. Edge and centre states for terminal branch lengths are defined by cell location at the end of the simulation, where edge cells are defined by being the most extreme cell on either the *X* or *Y* spatial axis for each row and column, respectively, or within one cell of this boundary. The fraction of the lineage time spent on the edge is determined by averaging across all lineage node states weighted by time tree branch lengths.

#### Continuous space model

To probe the robustness of SDevo to more complex selective events and higher dimensions, we implemented an additional set of simulations in the physics-based cellular simulator, PhysiCell^70^. Briefly, PhysiCell is an open-source, agent-based model implemented in C++ in which cell movement is governed by biomechanical interactions among cells. To simulate boundary-driven growth, we created a PhysiCell instance in which cells are only able to divide when under low mechanical pressure, using the cell-state variable, simple_pressure. As a result, similar to the Eden model, most cell division is restricted to the tumour periphery, or to cells with adjacent space created by the recent death of a neighbouring cell. Cells initially divide at a rate we arbitrarily set to 1, except when above the pressure threshold, *τ*, in which case they divide at rate 0. We also explored a sigmoidal relationship between pressure and birth, where the birth rate *b* = 1 − (1 + exp(−5(pressure − *τ*)))^−1^. Cells die at rate *d*, regardless of their pressure status. To simulate selection, during each cell division, a daughter cell can acquire a driver mutation conferring a 10% fitness advantage^95^ with probability *μ*_driver_, which acts multiplicatively (that is, a cell with two drivers has a 21% faster growth rate than one with 0)^96^. Tumours are grown to a final size of *N* extant cells, of which *n* are sampled. After the simulation, a Poisson-distributed number of neutral mutations is augmented to each cell division with *λ* = *μ*_passenger_. Using the continuous space model, we investigated all pairwise combinations of 2D and 3D, neutral and selective scenarios, and ran 25 tumour simulations for each combination of parameters (*τ* = 1, *d* = (0, 0.1, 0.2,…0.8), *μ*_passenger_ = 1, *n* = 100), except for 3D selection, where we simulated *d* = (0, 0.2, 0.6, 0.8) with 10 tumours each. For the 2D models, *N* = 10, 000, and for the 3D model, *N* = 15, 000. For the selective model, *μ*_driver_ = 0.01, and for the neutral models, *μ*_driver_ = 0. Note, we used a value of *μ*_driver_ well above expected rates of driver mutations (~10^−5^)^92^ to conservatively test SDevo in an extreme case of selection. To probe SDevo’s performance when cellular constraint is reduced by migration instead of cell death, we performed 10 simulations at *d* = 0.2, where cells migrate at 0, 0.5, 1, 1.5 or 2μm min^−1^ at an angle drawn from [0, 2π] and updated on average each minute (all other parameters as above). To probe SDevo’s robustness under a sigmoidal relationship between pressure and birth rate, we ran 10 simulations with *d* = (0, 0.2, 0.4, 0.6), and all other parameters as above. One outlier in the 3D boundary-driven growth simulations was removed due to convergence on a local optimum. Ground truth edge and centre birth rates were determined by first classifying cells as within 10 μm (approximately 1 cell width) of the tumour periphery as edge, and those more than 10 μm from the edge as centre. The average birth rate was computed separately within each of those classes over multiple discrete time points (10–40, depending on the overall rate of tumour growth) and combined by a weighted average according to the number of cells at each time point. Cells under too much pressure to divide at the sampled time (simple_pressure > *τ*) were calculated as having an instantaneous birth rate of 0.

#### Sampling procedures

2D simulations were sampled by maximizing the distance between sampled single cells in physical space (diversified sampling). This ensures that a sufficient number of edge and centre classified cells were sampled and that sampled cells were not clustered. Bulk punch biopsy sampling was mimicked by choosing a centre cell and a target of eight cells immediately surrounding that were grouped into a single punch. Punches were iteratively drawn and shifted if they overlapped with a previously punched group of cells. Sampling ended when the target number of punches was reached (50 punches) or sampling was no longer possible without significant overlap. Punch sequences were generated using all mutations above a cellular fraction cutoff of 0.3. 3D sampling was approximated by taking five simulated slices through the tumour *z*-plane at 2/8, 3/8, 4/8, 5/8 and 6/8 of the range of the *z* values of a given tumour. Within each slice, cells were sampled to maximize the inter-cell distance, as described above, and the number of cells per slice was proportional to the number of cells in the slice relative to the number of cells across all slices.

### Multi-type birth–death models of boundary-driven growth

The birth–death process describes how lineages duplicate (birth), die (death) and are sampled (where samples are tips on a phylogenetic tree)^97^. The multi-type birth–death model extends this by considering birth, death and sampling to occur in different states (sometimes also referred to as different sub-populations, traits or types) and how lineages jump between these states. The rates of birth, death and sampling vary depending on the state of a lineage. For the case of boundary-driven growth, we model a two-state process, with one state denoting cells in the centre of the tumour and the other state denoting cells on the edge of the tumour.

### Posterior probability

To perform Bayesian inference, we define the posterior probability *P*(*T*, *σ*, *θ*∣*D*) of the timed phylogenetic tree *T*, the evolutionary model and parameters (*σ*), and the population model and parameters *θ*, given the data, *D*. This posterior probability is typically expressed as:

1 $$P(T,\sigma ,\theta | D)=\frac{P(D| \sigma ,T)P(T| \theta )P(\sigma )P(\theta )}{P(D)}.$$

In the case of the state-dependent multi-type birth–death model, we cannot assume the tree likelihood (*D*∣*σ*, *T*) and the tree prior *P*(*T*∣*θ*) to be independent, as the rate of evolution directly depends on the population model. In other words, how fast evolution happens on a lineage depends directly on the state of that lineage. We therefore define ${{{\mathcal{H}}}}$ as a mapped state transition history that contains a random mapping of state change events given a set of parameters *θ* of the multi-type birth–death model. We then define the tree likelihood as $P(D| \sigma ,\theta ,T,{{{\mathcal{H}}}})$. Additionally, we say that instead of computing *P*(*T*∣*θ*) directly, we only compute the tree prior for one realization of the state transition history, that is, $P(T,{{{\mathcal{H}}}}| \theta )$. The posterior probability then becomes:

2 $$P(T,{{{\mathcal{H}}}},\sigma ,\theta | D)=\frac{P(D| \sigma ,\theta ,T,{{{\mathcal{H}}}})P(T,{{{\mathcal{H}}}}| \theta )P(\sigma )P(\theta )}{P(D)}.$$

Performing Markov chain Monte Carlo (MCMC) inference to characterize this posterior probability distribution would require integrating over all transition histories ${{{\mathcal{H}}}}$ using MCMC. This is overall incredibly slow and limits the application of the method. Instead, we formally integrate over all possible histories ${{{\mathcal{H}}}}$, to get the following posterior probability:

3 $$P(T,\sigma ,\theta | D)=\frac{{\int}_{{{{\mathcal{H}}}}}P(D| \sigma ,T,{{{\mathcal{H}}}}){\int}_{{{{\mathcal{H}}}}}P(T,{{{\mathcal{H}}}}| \theta )P(\sigma )P(\theta )}{P(D)}.$$

$P(T| \theta )={\int}_{{{{\mathcal{H}}}}}P(T,{{{\mathcal{H}}}}| \theta )$ is computed as described in ref. ^61^, which is achieved by treating the states of lineages probabilistically instead of discretely.

Last, we set ${\int}^{{{{\mathcal{H}}}}}P(D| \sigma ,T,{{{\mathcal{H}}}})=E\left.\right[P(D| \sigma ,\theta ,T,{{{\mathcal{H}}}})=P(D| \sigma ,\theta ,T,E[{{{\mathcal{H}}}}])$, with $E[{{{\mathcal{H}}}}]$ being the expected/average state transition history, which contains, for each lineage *i* in the phylogeny, its expected time spent in each state *s*. This leaves us with:

4 $$P(T,\sigma ,\theta | D)=\frac{P(D| \sigma ,\theta ,T,E[{{{\mathcal{H}}}}])P(T| \theta )P(\sigma )P(\theta )}{P(D)}.$$

### Modelling birth-dependent evolution

In order to model different rates of evolution for different states, we first compute the expected time each lineage in the phylogenetic tree *T* spent in each state. To do so, we use a stochastic mapping approach related to those described in refs. ^98,99^. We first compute the probability ${g}_{s}^{\,i,b}$ of each lineage *i* in the phylogenetic tree being in any possible state *s* over time *t* from the tips to the root (denoted with *b* for backwards in time) as described in ref. ^61^. These state probabilities are conditional only on events that occurred more recently than *t* and therefore not on all events in the phylogeny. During this backwards propagation, we keep track of the time-dependent transition matrix *Q*(*t*)^*i*^ that describes the rate of probability flow between any two states at time *t* due to state transitions or birth events between states. As a result, once we reach the root, ${g}_{s}^{\,i,b}$ contains all events in the phylogeny and is therefore equal to ${g}_{s}^{\,i,\,f}$, that is, the forward in time probability *f* of lineage *i* being in state *s*.

Following ref. ^100^, we first define ${q}_{ab}^{i}$ as:

$${q}_{ab}^{i}={\mu }_{ab}\frac{{g}_{b}{(t)}_{a}^{i}}{{g}_{b}{(t)}_{b}^{i}}$$

with *μ*_*ab*_ being the rate of state change due to state transitions or cross-birth events between states *a* and *b*.

We then compute the probabilities of any lineage being in any possible state conditional on all events in the phylogeny ${g}_{s}^{i,f}$ forwards in time as:

$$\frac{{\mathrm{d}}{g}_{s}^{\,i,\,f}}{{\mathrm{d}}t}=\mathop{\sum }\limits_{a=1}^{{\mathrm{states}}}\left({q}_{as}^{i}{g}_{a}^{\,i,\,f}-{q}_{sa}^{i}{g}_{s}^{\,i,\,f}\,\right).$$

By keeping track of the forward probabilities ${g}_{s}^{i,f}$ on each lineage, we can then compute the expected time ${t}_{s}^{i}$ that lineage *i* spends in any of the possible states *s*. The values for ${t}_{s}^{i}$ make up the entry for $E[{{{\mathcal{H}}}}]$ in the posterior distribution (equation (4)). We then say that *c*_*s*_ is the rate of evolution, that is, the clock rate, of a lineage in state *s*. Next, we compute the average rate of evolution on branch *i*, *c*^*i*^, as:

$${c}^{i}=\mathop{\sum }\limits_{s}^{{\mathrm{states}}}{t}_{s}^{i} \times {c}_{s}.$$

At each replication, an error in copying the genetic material of a cell can occur. These errors tend to be more likely in cancer cells, where cellular control mechanisms are often faulty. Phylogenetic methods typically assume the evolutionary processes to be independent of population processes, such as cell replication. To model mutations happening at birth events, we assume that the birth rate *b*_*s*_ in state *s* and the clock rate in state *s* are proportional such that *c*_1_ = *c*_avg_*b*_1_, *c*_2_ = *c*_avg_*b*_2_,…, *c*_*n*_ = *c*_avg_*b*_*n*_.

### Implementation

We implemented the multi-type birth–death model with state-dependent clock rates as an addition to the Bayesian phylogenetics software BEAST 2 (https://github.com/nicfel/SDevo). SDevo depends on BDMM-Prime v0.0.30 (https://github.com/tgvaughan/BDMM-Prime) to compute the tree prior *P*(*T*∣*θ*) and is built in Beast v2.6.6. To model mutations occurring at cell division, we set the relative rate of evolution in the different compartments (edge and centre) to be proportional to the birth rates in these compartments. The implementation itself does not explicitly require this assumption and the relative rates of evolution can also be treated as a distinct parameter in the inference. All SDevo analyses were performed using SDevo v0.0.2. SDevo can be installed through the interface BEAUti.

### Validation

To validate the implementation, we perform a well-calibrated simulation study. In it, we simulate phylogenetic trees under a two-state birth–death model in which we assume the rate of evolution to be proportional to the birth rate in either compartment. We randomly sample the birth, death and transition rates from the prior distribution, while fixing the sampling rate to 0.001, and then simulate a phylogenetic tree using MASTER^101^. We then simulate genetic sequences on top of the phylogenetic trees using different rates of evolution depending on the lineage’s compartment. Next, we infer the birth, death and transition rates from the genetic sequences and show that the 95% HPD interval covers the truth in 95% of the 100 runs (Extended Data Fig. 2).

### SDevo application to simulated tumours

We applied SDevo to outputs of the Eden and PhysiCell simulations generated as described above. For each simulated tumour we calculated clock rate (mutations/tree length/sequence length) and edge and centre sampling rates (sampled/alive cells). We set exponential priors on birth, death and transition rates. Full parameterization can be reproduced from XML templates. MCMC chains were run to convergence. We used only chains that had a minimum effective sample size for birth rate parameters greater than 200 for analysis. We also excluded rare (*n* < 5) cases that converged to local optima. We summarized the output posterior distributions by mean and 95% HPD intervals. We further inferred maximum clade credibility (MCC) trees with median heights using BEAST 2.6.2 TreeAnnotator^62^. TreeAnnotator also gives posterior state probabilities for each MCC internal node.

### SDevo application to HCC tumours

To apply SDevo to the HCC data, we labelled punches based on edge/centre state labels as published by ref. ^40^, Table S8 (reproduced in Fig. 5a,e). For alternative states (Extended Data Fig. 7a,e), we labelled punches as edge if they were located within approximately 10% ( ~2 mm for Tumour 1 and ~1.5 mm for Tumour 2) of the tumour diameter from the schematic boundaries. Slices were reported to be from tumour hemispheres. Assuming a 0.2 mm slice thickness, we estimated that slices Tumour 1Z and Tumour 2Z fell within the boundary region. The original amplicon genotyping panel artificially increases the apparent diversity within some clones relative to others, so to avoid incorporating this bias into the model, we used only whole-genome sequenced punches. Ref. ^40^ identified a large number of SNVs (254,268 for Tumour 1 and 142,032 for Tumour 2). To reduce computational requirements and improve convergence, we generated input pseudo-sequences by randomly subsampling 25,000 variable sites. We summarized results across three independent subsamples for each tumour. We called presence or absence of a variant at each site based on a VAF cutoff of 0.05. VAF histograms displayed single-peaked distributions characteristic of a single major clone per sample, with the exception of tumour sample T1L13 (Extended Data Fig. 9). To ensure Tumour 1 results were not driven by over-counting mutations across multiple subclones of T1L13, we repeated the analysis excluding this sample and found quantitatively similar results (Extended Data Fig. 8).

We use a ${{{\rm{GTR}}}}+{\Gamma }_{4}$ site model, a fixed clock rate of 0.3 (units are arbitrary as we only use sites that are variable relative to healthy cells) and estimate sampling proportion (uniform prior). We use log-normal priors for birth (mean = 20, *S* = 0.5) and death rates (mean = 15, *S* = 0.5), with S denoting the standard deviation of the log-transformed distributions. We used an exponential prior for the edge-to-centre transition rate (mean = 1). Note that these units are also arbitrary and are not calibrated to clinical time. In applying SDevo to these tumours, we constrain the parameter space in several ways to adapt to having relatively few samples, only a single observed time point and unknown sampling proportion. (1) We assume unidirectional transition so that cells can only move from edge to centre but not vice versa. As we only have a few observed state transition events, the transition rates would otherwise be relatively poorly informed. (2) We set priors on mean birth, death and transition rates across the two states. Birth and death priors are identical across both states, while transition rates priors are asymmetrical to inform unidirectional transition and enable convergence in a complex parameter space. Full parameterization can be found in the XML template. We combined posterior estimates across three independent runs for each tumour. We inferred MCC trees with ancestral state reconstructions with TreeAnnotator. In addition to the SDevo-inferred trees and parameters, we also generated maximum likelihood trees using FastTree^102^ and Augur^103^ under a Jukes–Cantor model for each tumour using all reported variable sites. Homoplastic sites contributed to lower support for one node in the maximum likelihood tree of Tumour 1 (Fig. 5b) and we masked homoplastic sites to enable convergence in Tumour 1 and Tumour 2 SDevo inferences. Homoplastic sites represented <1% (Tumour 1) or 6% (Tumour 2) of all sites across all tumour samples. In Tumour 2, more than two-thirds of homoplasies were between two edge-associated punches (Z1 and Z13) potentially pointing to subclonal mixing, which is supported by their proximal spatial locations. The remainder of homoplasies in Tumour 2 and all of the homoplasies in T1 were evenly distributed across punches. As a result, the removal of homoplasies did not act to bias branch lengths across the tree, with the exception of T2Z1 and T2Z13. As these punches are on the edge of the tumour, this masking should a priori result in lower estimated birth rates on the edge and thus conservatively bias the results towards a more equal birth rate between edge and centre.

### Reporting summary

Further information on research design is available in the Nature Portfolio Reporting Summary linked to this article.

## Supplementary information

Reporting Summary

## Data Availability

Data required to reproduce analyses are available at https://github.com/blab/spatial-tumor-phylodynamics, including variant allele frequencies and input BEAST 2 XML files. Raw sequencing data are publicly available (GSA-Human: HRA000188) as published by ref. ^40^. SNVs used for the HCC analysis are provided in a de-identified format on GitHub. Please cite ref. ^40^ if using these data.

## Code availability

Custom scripts were used for simulation studies and data analyses. All code to generate figures is publicly available. Scripts to replicate analyses and figures are available at https://github.com/blab/spatial-tumor-phylodynamics, including a local R package tumortree (https://github.com/blab/spatial-tumor-phylodynamics/tree/main/tumortree), which can be installed to build trees from the simulation outputs. SDevo is built as a BEAST 2 package and can be installed from https://github.com/nicfel/SDevo. The source code to run spatially constrained PhysiCell simulations and generate trees can be found here at https://github.com/federlab/PhysiCellTrees. All other packages used for analysis and visualization are also open-source^104,105^.
