# Supplementary material for: State-dependent evolutionary models reveal modes of solid tumour growth
Source: Nat Ecol Evol. 2023 Mar 9;7(4):581–96. doi: 10.1038/s41559-023-02000-4 (PMC10089931; doi:10.1038/s41559-023-02000-4)
Supplement: Supplementary file 1 — Reporting Summary [file 41559_2023_2000_MOESM1_ESM.pdf]

Corresponding author(s): Maya A. Lewinsohn
Nicola F. Mueller
Alison F. Feder

Last updated by author(s): 01/06/2023

Reporting Summary

Nature Portfolio wishes to improve the reproducibility of the work that we publish. This form provides structure for consistency and transparency in reporting. For further information on Nature Portfolio policies, see our Editorial Policies and the Editorial Policy Checklist.

Statistics

For all statistical analyses, confirm that the following items are present in the figure legend, table legend, main text, or Methods section.

n/a	Confirmed	
☐	☒	The exact sample size (*n*) for each experimental group/condition, given as a discrete number and unit of measurement
☐	☒	A statement on whether measurements were taken from distinct samples or whether the same sample was measured repeatedly
☐	☒	The statistical test(s) used AND whether they are one- or two-sided<br>*Only common tests should be described solely by name; describe more complex techniques in the Methods section.*
☒	☐	A description of all covariates tested
☐	☒	A description of any assumptions or corrections, such as tests of normality and adjustment for multiple comparisons
☐	☒	A full description of the statistical parameters including central tendency (e.g. means) or other basic estimates (e.g. regression coefficient) AND variation (e.g. standard deviation) or associated estimates of uncertainty (e.g. confidence intervals)
☒	☐	For null hypothesis testing, the test statistic (e.g. *F*, *t*, *r*) with confidence intervals, effect sizes, degrees of freedom and *P* value noted<br>*Give P values as exact values whenever suitable.*
☐	☒	For Bayesian analysis, information on the choice of priors and Markov chain Monte Carlo settings
☒	☐	For hierarchical and complex designs, identification of the appropriate level for tests and full reporting of outcomes
☒	☐	Estimates of effect sizes (e.g. Cohen's *d*, Pearson's *r*), indicating how they were calculated

Our web collection on statistics for biologists contains articles on many of the points above.

Software and code

Policy information about availability of computer code

Data collection	No data was collected as part of this study.
Data analysis	Custom scripts were used for simulation studies and data analyses. Scripts to replicate analyses and figures are available at https://github.com/blab/spatial-tumor-phylodynamics, including a local R package tumortree 0.0.1 (https://github.com/blab/spatial-tumor-phylodynamics/tumortree), which can be installed to build trees from the simulation outputs. SDevo v0.0.2 was built using Beast v2.6.6 and BDMM-Prime v0.0.30, and the source code is available here: https://github.com/nicfel/SDevo. The source code to run spatially-constrained PhysiCell 1.9.1 simulations and generate trees can be found here: https://github.com/federlab/PhysiCellTrees. TreeAnnotator v2.6.2 and LogCombiner v2.6.2, which are part of the BEAST2 platform (https://www.beast2.org/), were used to process MCMC logs. R 4.0.4 was used for analyses and packages include tidyverse 1.3.2, beastio 0.3.3, ape 5.6.2, phangorn 2.7.1, treeio 1.17.2, HDInterval 0.2.4, and coda 0.19.4. MCC trees were plotted with ggtree 3.5.2 and other visualizations were created in ggplot2 3.3.6. Matlab scripts were run using MATLAB_R2019b.

For manuscripts utilizing custom algorithms or software that are central to the research but not yet described in published literature, software must be made available to editors and reviewers. We strongly encourage code deposition in a community repository (e.g. GitHub). See the Nature Portfolio guidelines for submitting code & software for further information.

Data

Policy information about availability of data

All manuscripts must include a data availability statement. This statement should provide the following information, where applicable:

- Accession codes, unique identifiers, or web links for publicly available datasets
- A description of any restrictions on data availability
- For clinical datasets or third party data, please ensure that the statement adheres to our policy

The BEAST2 input xml files for all hepatocellular carcinoma analyses in this manuscript, as well as the files used to post process these analyses are available from https://github.com/blab/spatial-tumor-phylodynamics. The xml files include the sequence data and exact input specification of the BEAST2 analyses performed in this manuscript. Data analyzed in this study are also publicly available as part of a published study by Li et al (MBE 2021, https://academic.oup.com/mbe/article/39/1/msab335/6440067, GSA-Human: HRA000188) and should be cited for any future use of derived data. Processed data derived from this study are available at https://github.com/blab/spatial-tumor-phylodynamics.

Human research participants

Policy information about studies involving human research participants and Sex and Gender in Research.

Reporting on sex and gender	does not apply
Population characteristics	does not apply
Recruitment	does not apply
Ethics oversight	does not apply

Note that full information on the approval of the study protocol must also be provided in the manuscript.

Field-specific reporting

Please select the one below that is the best fit for your research. If you are not sure, read the appropriate sections before making your selection.

☒ Life sciences ☐ Behavioural & social sciences ☐ Ecological, evolutionary & environmental sciences

For a reference copy of the document with all sections, see nature.com/documents/nr-reporting-summary-flat.pdf

Life sciences study design

All studies must disclose on these points even when the disclosure is negative.

Sample size	For hepatocellular cancer dataset, Tumor 1 had N=16 punches and Tumor 2 had N=9 punches. For simulation studies, the number of samples varied from a minimum of 10 to a maximum of 100 sampled tips.
Data exclusions	For simulation studies, only MCMC analyses that had reached an effective sample size (ESS) threshold of 200 were included. Rare (<5) cases of convergence to a local optima were also excluded and are noted in the methods. For the hepatocellular carcinoma datasets, all punch biopsies were included for main figure analyses. In Figure S10, Tumor 1 is re-analyzed excluding punch T1L13.
Replication	Publicly available sequencing data were used, as such, the sequencing itself can't be replicated. For hepatocellular carcinomas, three independent MCMC runs per formed per analysis by randomly subsampling the dataset 3 times for each analysis.
Randomization	For hepatocellular carcinoma datasets, we generated input pseudo-sequences by randomly subsampling 25,000 variable sites. We generated three independent subsets per tumor.
Blinding	does not apply, as we analyzed publicly available sequence data.

Reporting for specific materials, systems and methods

We require information from authors about some types of materials, experimental systems and methods used in many studies. Here, indicate whether each material, system or method listed is relevant to your study. If you are not sure if a list item applies to your research, read the appropriate section before selecting a response.

Materials & experimental systems

n/a	Involved in the study
☒	☐ Antibodies
☒	☐ Eukaryotic cell lines
☒	☐ Palaeontology and archaeology
☒	☐ Animals and other organisms
☒	☐ Clinical data
☒	☐ Dual use research of concern

Methods

n/a	Involved in the study
☒	☐ ChIP-seq
☒	☐ Flow cytometry
☒	☐ MRI-based neuroimaging
